# Heat knockdown resistance and chill‐coma recovery as correlated responses to selection on mating success at high temperature in *Drosophila buzzatii*

**DOI:** 10.1002/ece3.6032

**Published:** 2020-02-06

**Authors:** Leonel Stazione, Fabian M. Norry, Federico H. Gomez, Pablo Sambucetti

**Affiliations:** ^1^ Departamento de Ecología Genética y Evolución Facultad de Ciencias Exactas y Naturales Universidad de Buenos Aires Buenos Aires Argentina; ^2^ Instituto de Ecología Genética y Evolución (IEGEBA) CONICET‐Universidad de Buenos Aires Buenos Aires Argentina

**Keywords:** experimental evolution, mating success, thermotolerance, *trade‐off* association

## Abstract

Reproduction and related traits such as mating success are strongly affected by thermal stress. We tested direct and correlated responses to artificial selection in replicated lines of *Drosophila buzzatii* that were selected for mating success at high temperature. Knockdown resistance at high temperature (KRHT) and chill‐coma recovery (CCR) were tested as correlated selection responses. Virgin flies were allowed to mate for four hours at 33°C in three replicated lines (S lines) to obtain the selected flies and then returned at 25°C to lay eggs. Other three replicated lines were maintained at 25°C without any selection as control (C lines). After 15 selection generations, KRHT and CCR were measured. Both traits were assessed in flies that did not receive any hardening pretreatments as well as in flies that were either heat or cold hardened. Thermotolerance traits showed significant correlated responses with higher KRHT in S than in C lines, both with a heat‐hardening pretreatment and without a heat‐hardening pretreatment. CCR time was longer in S than in C lines both with a cold‐hardening pretreatment and without a cold‐hardening pretreatment. Hardening treatments improved both KRHT and CCR in all cases excepting KRHT in C lines. Overall, KRHT and CCR showed an antagonistic pattern of correlated responses to our selection regime, suggesting either pleiotropy or tightly linked trait‐specific genes partially affecting KRHT and CCR.

## Graphical abstract

Artificial selection for mating success at high temperature in replicated lines of *Drosophila buzzatii* was performed. Knockdown resistance to high temperature (KRHT) and chill‐coma recovery (CCR) were tested for correlated selection responses. KRHT and CCR showed an antagonistic pattern of correlated responses to our selection regime, suggesting an indirect trade‐off.

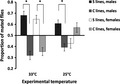

## 1 INTRODUCTION

High temperature can affect the reproduction, abundance, and distribution of species in contemporaneous wild environments under heat stress (Franks & Hoffmann, 2012; Hoffmann & Sgrò, 2011). In addition, the ability of individuals to respond to adverse environmental conditions such as thermal stress is highly relevant for adaptation to climate changes including global warming (Borda, Sambucetti, Gomez, & Norry, 2018; Deutsch et al., 2008; Franks & Hoffmann, 2012; Huey et al., 2012; Kellermann, Heerwaarden, Sgrò, & Hoffmann, 2009; Kellermann et al., 2012; Kingsolver et al., 2011; Rebaudo & Rabbi, 2018; van Heerwaarden, Kellermann, & Sgrò, 2016). Diverse fitness‐related traits, including mating success at stressing temperatures, can be direct phenotypic targets of selection for thermal adaptation (Hoffmann, Sørensen, & Loeschcke, 2003; Kellermann et al., 2009; Sambucetti & Norry, 2015).

Mating success is an important component of reproductive fitness (Brooks & Endler, 2001). Thermal stress affects the level of organism's activity and can result in failure to mate (Miwa, Koganezawa, & Yamamoto, 2018). Therefore, mating success at elevated temperature can be a direct target of selection for adaptation to environmental temperature. In warm environments, mating success can evolve in response to temperature (Dolgin, Whitlock, & Agrawal, 2006; Fasolo & Krebs, 2004; Sambucetti & Norry, 2015), where the ability to mate under heat stress should be determinant for reproductive success. Thus, selection for mating success under elevated temperature could be an important component of selection for adaptation to warm environmental conditions.

In insects, knockdown resistance to high temperature (KRHT) and chill‐coma recovery (CCR) represent ecologically relevant phenotypes for thermal adaptation in the adult (reproductive) stage of the life cycle (e.g., Gibert, Moreteau, Pétavy, Karan, & David, 2001; Hoffmann, Anderson, & Hallas, 2002; Hoffmann et al., 2003; Huey, Crill, Kingsolver, & Weber, 1992; Morgan & Mackay, 2006; Norry, Gomez, & Loeschcke, 2007; Rako, Blacket, McKechnie, & Hoffmann, 2007). In *D. melanogaster*, KRHT and CCR show opposing latitudinal clines attributable to thermal adaptation at the eastern coast of Australia (Hoffmann et al., 2002). KRHT and CCR appeared to be negatively correlated in a quantitative trait locus (QTL) affecting both traits in the middle of chromosome 2 in *D. melanogaster*, indicating either linkage or pleiotropy (Morgan & Mackay, 2006; Norry et al., 2007; Norry, Scannapieco, Sambucetti, Bertoli, & Loeschcke, 2008; see also Loeschcke, Kristensen, & Norry, 2011 for a field assay). In this species, another genome region involving 3‐R‐Payne was also implicated in a trade‐off between cold resistance and heat resistance (Anderson, Collinge, Hoffmann, Kellett, & McKechnie, 2003). However, artificial selection for KRHT had no correlated responses on CCR in *D. buzzatii* (Sambucetti, Scannapieco, & Norry, 2010). In addition, selection for CCR had no correlated responses on KRHT in *D. buzzatii* (Bertoli, Scannapieco, Sambucetti, & Norry, 2010). Similar results of no trade‐off association in correlated selection responses were obtained for CCR and survival after heat stress in *D. melanogaster* (Gerken, Mackay, & Morgan, 2016; Mori & Kimura, 2008). Nevertheless, *Drosophila* and other insects can constantly adapt to their surrounding environment in the field, where heat resistance can be negatively correlated with cold resistance (Condon et al., 2015; Overgaard & Sørensen, 2008). It is still possible that selection on a direct target of adaptation to warm environments, including mating success at high temperature, can affect CCR detrimentally, either by pleiotropy or by linkage.

Here, we used artificial selection on mating success at high temperature to test for direct and correlated responses to thermal sexual selection in *Drosophila buzzatii*. Artificial selection is a useful tool to test not only the direct response to selection but also any possible correlated responses in other traits. To do this, we mass crossed two natural populations representing the extremes of an altitudinal cline to establish a base population in which clinal traits should be expected to segregate substantial variation. This could allow us to detect any possible correlated responses that were no evident when using a base population established from only one of the extremes of the cline (e.g., Bertoli et al., 2010; Sambucetti et al., 2010). It is well‐known that stress resistance usually increases by previous exposures to sublethal mild stress, a phenomenon called hardening effect (Bowler & Terblanche, 2008; Hoffmann et al., 2003; Loeschcke & Hoffmann, 2007; Stazione, Norry, & Sambucetti, 2019). Therefore, we tested both KRHT and CCR with a hardening pretreatment and without a hardening pretreatment as possible correlated responses to mating selection at high temperature. Two main aims were addressed. First, we tested whether or not mating success under heat stress increases by artificial selection (i.e., heritability hypothesis for mating success). Second, we also tested correlated responses on both KRHT and CCR, given their well‐known ecological relevance for thermal adaptation and their possible trade‐off associations as mentioned above (i.e., trade‐off hypothesis).

## 2 MATERIALS AND METHODS

### 2.1 Fly stocks

Our base population was set up from a mass crossing between samples from two natural populations from the extremes of a previously studied altitudinal cline from northwestern Argentina (Norry, Sambucetti, Scannapieco, & Loeschcke, 2006; Sambucetti, Loeschcke, & Norry, 2006; Sørensen, Norry, Scannapieco, & Loeschcke, 2005). In April 2009, *D. buzzatii* flies were collected from two populations of relatively low and high altitudes, at Chumbicha (401 masl; 28°53′S, 65°16′W) and Quilmes (1855 masl; 26°28′S, 66°02′W), respectively. Forty laboratory‐reared flies from each population were placed in 125‐ml glass culture bottles containing 40 ml of a potato‐based culture medium fully described in Gomez, Stazione, Sambucetti, and Norry (2020), hereafter standard cultures. Ten such cultures were set up, with 20 virgin flies of each sex from each population. To control for any possible effects of laboratory adaptation in the response of our thermal selection regime (Orozco‐Terwengel et al., 2012), cultures were maintained for 10 generations before the mass crossing between populations, and the mass‐crossed population was maintained for other five generations before the start of thermal selection.

Experimental individuals for both selection and control regimes were virgin flies at the F5 generation from the crossing between the above‐mentioned laboratory cultures (i.e., the G15 laboratory generation) that emerged from standard cultures at 25 ± 1°C under a 12:12‐hr L:D cycle. Virgin flies were split into two sets. One set were the control lines, in three replicates, denoted C_1_, C_2_, and C_3_. The other set corresponds to artificially selected flies for mating success at high temperature, in three replicates, denoted S_1_, S_2_, and S_3_. Each replicated line was maintained over four standard culture bottles at 25 ± 1°C on a 12:12‐L:D cycle.

### 2.2 Selection regime

Flies for each selection generation were obtained from 4 to 5 standard bottles per stock with 30 males plus 30 females per bottle. Flies were allowed to lay eggs for four days and after that removed from the bottles. Virgin flies emerging from these bottles (collected within 5 hr) were sexed under slight CO_2_ anesthesia and placed in standard vials with fresh food. Approximately 100 virgin females plus 100 virgin males of 3–4 days of age were placed in 500‐ml glass bottle (35 × 9 cm) with 50 ml of fresh culture medium (instant mashed potatoes with water, nipagin, and yeast as culture medium) at 33°C in a walk‐in incubator for 4 hr to allow copulation for each replicated S line. Each female was then transferred separately to a new vial with 2 ml of our standard culture medium for five days at 25°C, resulting so in a moderate larval density from each inseminated female, and their offspring were subsequently collected in mass from all vials as progenitors of the next generation. These mass‐collected flies were placed at a density of 20 females plus 20 males into new bottles containing 40 ml of culture medium. Thus, larval density in vials versus. bottles was relatively controlled by keeping similar proportion of culture medium (in mL) for the number of females (i.e., one female per 2 ml of culture medium in all cases). This procedure also allowed us to control for the number of females that effectively contributed to next generation. Mating success was thus selected at high temperature, and the selection regime was applied every other generation for 15 generations of artificial selection.

Control lines (C_1_, C_2_, C_3_) were obtained as S lines with the only difference that no selection for mating success at 33°C was applied, and only culture bottles (not vials) were used for rearing control flies. Each C line was maintained with at least 80 flies of each sex every generation, with 4 replicated culture bottles per replicated line for each generation, with similarly controlled larval density as for S lines.

### 2.3 Direct selection response on mating success

After the last generation of selection, mating success was scored at both 25 + 1°C and 33 + 1°C in a walk‐in incubator. Experimental flies were reared in standard bottles by placing 15 males plus 15 females per bottle per line, with 4 standard bottles per replicated line. Bottles were placed at 25°C under 12:12‐hr L:D period. Experimental virgin flies were obtained as described above. Mating success at each temperature (in incubator room at 25 + 1°C or 33 + 1°C) was evaluated in competition between replicates of both lines (S vs. C) in transparent plastic cages (20 × 12 × 10 cm), using a thin cloth net as lid. Two small dishes (2 cm diameter) containing standard‐potato‐based food plus yeast were placed inside each cage to stimulate courtship and mating. Forty individuals (1:1 sex ratio) from one S and one C replicate line were released within the mating cage, making a total of 80 individuals. These combinations were randomized so that all S replicated lines competed with all replicated C lines. All virgin flies from each line were marked 1 hr prior to the releasing within the cage, transferring flies to vials with 0.15 mg of fluorescent micronized dust and lightly shaken. Dust colors were randomly assigned to the different lines and changed between replications of the experiment.

Flies were observed during the next 4 hr between 1:00 p.m. and 5:00 p.m. Pairs in copula were collected from the cage by using an aspirator tube. Each pair was placed into an empty vial and frozen at −20°C before scoring the color of each fly at 10× magnification. Eight to ten mating cages were performed in total.

### 2.4 Heat knockdown resistance

Knockdown resistance to high temperature (KRHT) was measured in nonvirgin flies of both S and C lines for approximately 30 males and 30 females of 4–5 days of age, one generation after the G15 generation of selection. Both sexes were released into a knockdown tube (5 × 62.5 cm) at 37.5 ± 0.5°C. KRHT was scored every 30 s by using a collecting vial which was replaced every 30 s until the last fly in the column was knocked‐down. Measurements were done between 11:00 a.m. and 4:00 p.m., to avoid circadian variations and were performed two times at different days, and mean value of each independent measurement was averaged to obtain the final estimate of KRHT for each line. The trait was measured in flies that did not receive any heat‐hardening treatment as well as in flies that were exposed to a heat‐hardening pretreatment of 1 hr at 36°C, 22 hr before the KRHT measurement (Norry et al., 2008).

### 2.5 Chill‐coma recovery

Flies of 3 days of age were sexed under slight CO_2_ anesthesia. Thirty flies per sex and line were immediately transferred to empty vials and placed for 20 hr inside a thermal box containing melting ice (0°C) within a cold room at 4°C. After 20 hr, all flies (4 days old) were returned to 25°C. The CCR time was scored in sec for each fly as the time until an individual was able to stand on its legs. Measurements of CCR were done between 11:00 a.m. and 3:00 p.m., performed two times at different days and averaged for each line. CCR was measured in flies that did not receive cold‐hardening pretreatment as well as in flies that were cold pretreated by 2 hr at 4°C, 22 hr before the measurement.

### 2.6 Statistical analysis

Differences in mating success between S and C lines were tested with a chi‐square test separately for each sex and temperature (25 and 33°C) after pooling replicated lines for this analysis only. In addition, differences between S and C lines were further tested for each thermal treatment with an analysis of the deviances from a Gaussian distribution (best fitted distribution of the data) and logit link function in a generalized linear model (GLM). Analyses were performed separately for each temperature (25 and 33°C) using sex and line as fixed factors and replicates within line as a random factor. Number of matings was used as dependent variable. All analyses were implemented with InfoStat software (Di Rienzo et al., 2017), as an interface of the R platform version 3.4.1 (R Core Team, 2017) to estimate generalized linear models through GLM and GLMER procedures from the stats and lme4 libraries (Bates, Maechler, Bolker, & Walker, 2013).

KRHT and CCR were tested for between‐line variation with a three‐way ANOVA using sex, hardening pretreatment, and line (S and C) as fixed factors and replicate within line as a random factor. In addition, pretreatment effects on KRHT and CCR were also tested separately for each line with a two‐way ANOVA, using sex and line (with and without pretreatment) as fixed factors and replicates within line as a random factor. KRHT and CCR data were square root‐transformed to improve homogeneity of variances (Levene test, *p* > .05) and normality (Shapiro–Wilk test, *p* > .05).

## 3 RESULTS

**Figure 1:**
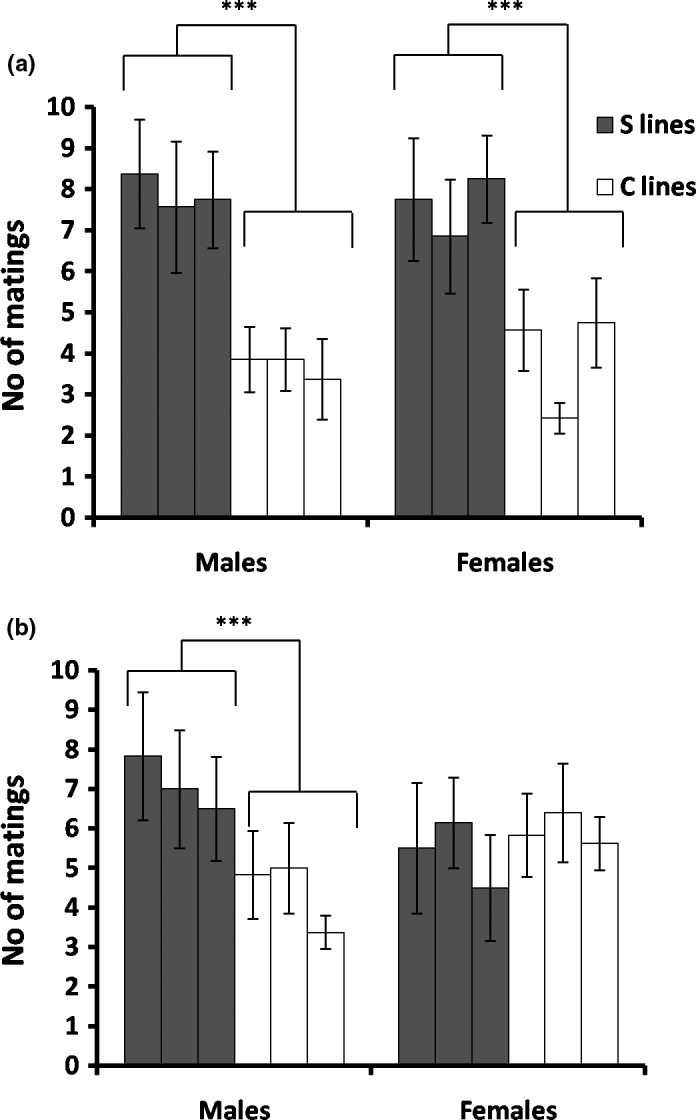
Number of mating flies (±SE) at 33°C (a) and 25°C (b) averaged over mating cages is shown for each sex in both S and C lines (***p < .001)

**Figure 2:**
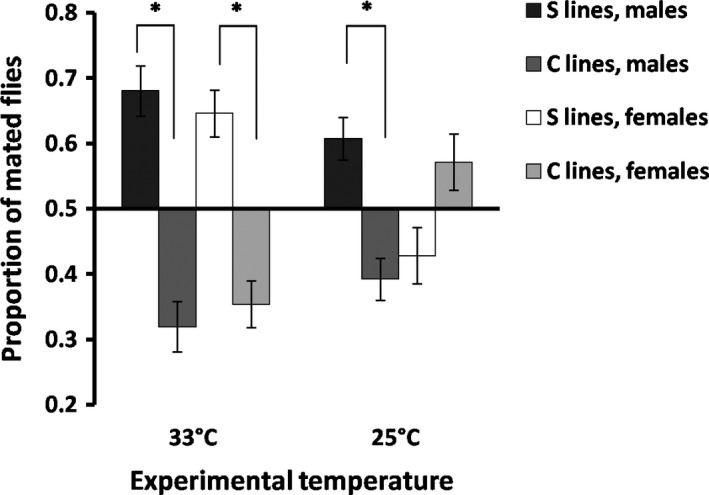
Proportion of mating flies relative to the number of copulates observed for the total of mating cages (±SE) both at 25 and 33°C is shown for each sex in by‐replicate‐pooled S and C lines. The 0.5 value in the *y*‐axis indicates no differences in the proportions between lines. Asterisks indicate significant differences (*p* < .05)

Mating success at elevated temperature significantly responded to our artificial selection regime (Figure 1). Proportion of mating flies in the competitive assay is shown in Figure 1 for pooled C lines (controls) as well as for S lines pooled over replicates at the G15 generation of selection. Mating success at 33°C was higher in S than in C lines (Figure 2; chi‐square test: $\chi_{1}^{2}$ = 26.25*** for males; $\chi_{1}^{2}$ = 24.9*** for females; ***p < .001). In addition, GLM did reveal significant differences in mating success between S and C lines at 33°C, with no difference between the sexes (Table 1; see also Table S1 for each replicate line). In contrast, at 25°C mating success was dependent on sex (Figure 2; chi‐square test: *χ* ^2^_1_ = 12.64*** for males; *χ* ^2^_1_ = 1.87 for females; ***p < .001). GLM showed that sex‐by‐line interaction was highly significant at this temperature (Table 1 and Supporting Information Table S1). Therefore, a simple‐effect analysis was performed to test mating success for each sex separately. In males, mating success was significantly higher in S than in C lines, while there were no significant differences between S and C females (Figure 1b. GLM with line as fixed factor: *F*_1_ = 13.21*** for males, *F*_1_ = 0.89, for females; ***p < .001).

**Table 1:** Generalized linear model (GLM) performed to test for the direct response to selection on mating success at high temperature, using line (S vs. C) and sex as fixed factors at each experimental temperature, 25 and 33°C

	33°C		25°C	
	*df*	*χ* ^2^	*df*	*χ* ^2^
Line	1	55.25***	1	3.87
Sex	1	0.03	1	0.25
Line × sex	1	0.3	1	10.76**

** *p * < .01;

*** *p * < .001.

**Figure 3:**
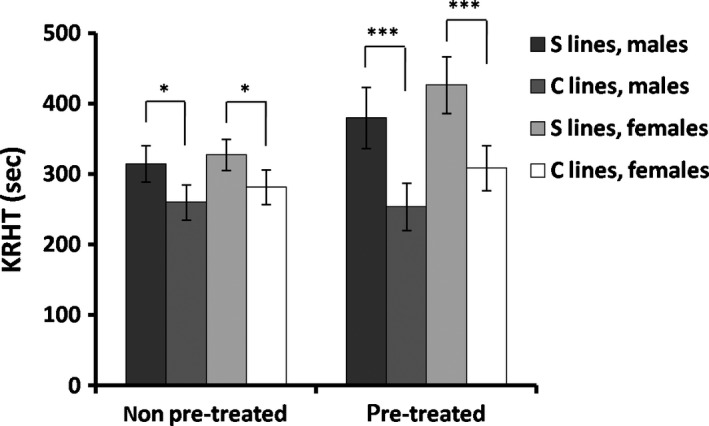
Mean values (± SE) of knockdown resistance to high temperature (KRHT) are shown for each sex with a heat‐hardening pretreatment and without a heat‐hardening pretreatment in both S and C lines (*p < .05; ***p < .001)

Knockdown resistance to high temperature (KRHT) was higher in S than in C lines, both with a heat‐hardening pretreatment and without a heat‐hardening pretreatment (Figure 3). This correlated response to our sexual selection regime was significant in three‐way ANOVA with no significant effects of heat hardening (Table 2; Supporting Information Figure S1; Table S2). However, when analyzing heat‐hardening separately for S and C lines, two‐way ANOVA revealed a significant effect in S lines, increasing KRHT (ANOVA with line [1] and sex [2] as fixed factors and replicate within line as a random factor: *F*_1, 423_ = 5.43* for [1]; *F*_1, 423_ = 0.86 for [2]; *F*_1, 423_ = 0.44 for [1] × [3]. *p < .05. Figure 3).

**Table 2:** ANOVAs on both KRHT and CCR, using S versus C lines, hardening pretreatment (HP) and sex as fixed factors, with replicate within line as a random factor

Factors	KRHT		CCR	
	*df*	*F*	*df*	*F*
Line	1	15.66***	1	84.59***
Hardening pretreatment (HP)	1	3.43	1	244.77***
Sex	1	3.04	1	22.63***
Line × HP	1	2.55	1	0.0002
Line × Sex	1	0.26	1	0.0004
HP × Sex	1	0.75	1	0.02
Line × HP × Sex	1	0.01	1	0.21
Error	838		1,058	

*** *p* < .001.

**Figure 4:**
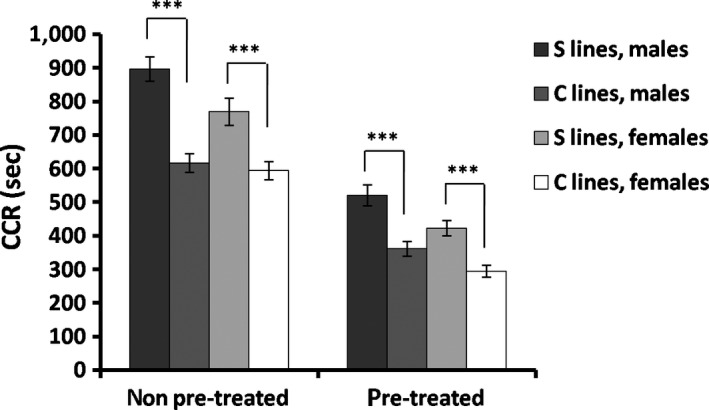
Mean values (± SE) of chill‐coma recovery (CCR) are shown for each sex with a cold‐hardening pretreatment and without a cold‐hardening pretreatment in both S and C lines (***p < .001)

Chill‐coma recovery (CCR) time was longer in S than in C lines both with a cold‐hardening pretreatment and without a cold‐hardening pretreatment (Table 2; Supporting Information Figure S2 for data in each replicated line see Supporting Information Table S3). This correlated response to our selection regime for increased mating success at high temperature was opposed to the trend observed for KRHT, suggesting a selection response negatively correlated between KRHT and CCR. ANOVA revealed a significant effect of cold‐hardening pretreatment both in S and C lines, decreasing chill‐coma recovery time (Figure 4; Table 2).

## 4 DISCUSSION

Mating success at elevated temperature strongly increased after 15 generations of artificial selection in our experimental population of *Drosophila buzzatii* (Figure 2). This result shows that mating success under heat stress can be a direct target of thermal selection. After understanding that the selection that operates on the advantage of certain individuals over others of the same sex and species in respect of reproduction is referred to as sexual selection (Darwin, 1871), the present results might be interpreted as a case of thermal sexual selection, with thermal tolerance being the target for differences in mating success. Importantly, this thermal sexual selection had a negative effect on cold resistance, increasing chill‐coma recovery time in both sexes (Figure 4), plus a positive effect on heat resistance, increasing KRHT (Figure 3). This clear‐cut result might suggest a *trade‐off* association between KRHT and CCR via mating success at high temperature.

In addition to the antagonistic responses observed for KRHT and CCR in the population selected, the results also revealed not only the presence of additive genetic variation but also a significant heritability for mating success at elevated temperature in both sexes. The role of selection for mating in adaptation of populations to new or changed conditions is a complex subject (reviewed in Candolin & Heuschele, 2008). Several studies showed that selection for mating under some specific environmental conditions is often relaxed under other environmental conditions (e.g., Candolin, Salesto, & Evers, 2007; Dolgin et al., 2006). Our results suggest that selection for mating at elevated temperature can also influence mating success at benign temperature but not in both sexes. Specifically, when mating was tested at 25°C, the difference between S and C lines was not only sex‐specific (in males only) but also much lower than in both sexes when mating success was tested at 33°C (Figure 1). Similar selective differences are typically found between the sexes at benign temperature (Singh & Punzalan, 2018). In addition, this finding is consistent with the well‐known hypothesis of stronger selection for mating in males than in females, as predicted from sexual selection theory (Andersson, 1994; Brooks & Endler, 2001; Darwin, 1871; Janicke, Häderer, Lajeunesse, & Anthes, 2016).

In *D. buzzatii*, selection for KRHT increased male mating success at high temperature, with no impacts on CCR (Sambucetti & Norry, 2015; Sambucetti et al., 2010). Here, we showed that selection for mating at elevated temperature can have important consequences on adaptation to both extremes of the thermal scale, by increasing not only KRHT but also CCR time, suggesting a possible *trade‐off* between heat resistance and cold resistance as a result of thermal selection in mating success. This is an important finding by suggesting otherwise hidden associations between complex traits of thermal adaptation, such as KRHT and CCR. As mentioned above, QTL studies showed a *trade‐off* association between KRHT and CCR for one QTL in the middle of chromosome 2 in diverse mapping populations in *D. melanogaster* (Morgan & Mackay, 2006; Norry et al., 2007, 2008). However, this *trade‐off* association was not apparent in experiments of artificial selection on either KRHT or CCR in *D. buzzatii* (Bertoli et al., 2010; Sambucetti et al., 2010; see Mori & Kimura, 2008 and Gerken et al., 2016 for similar results in *D. melanogaster*). Dominant gene action in a pleiotropic allele at high frequency might partially mask correlated selection responses (Defays, Gomez Fernandez, & Norry, 2009). In this study, we found a negative correlation between heat resistance and cold resistance as a correlated response to heat selection for mating success. In contrast to previous artificial selection studies in *D. buzzatii* where *trade‐off* associations between KRHT and CCR were not evident (Bertoli et al., 2010; Sambucetti et al., 2010), our base population in the preset study was established from a massive crossing between populations from the two extremes of a thermal cline. The broader initial genetic variation present in this design showed that antagonistic correlated responses between KRHT and CCR can evolve as result of artificial selection. We are aware that the correlated selection responses in KRHT and CCR could be the result of linkage rather than pleiotropy as linkage disequilibrium could be increased by crossing highland and lowland populations to construct our base population. However, this hybrid population was allowed to mate at random for 5 generations before the start of selection, allowing thus recombination, and much more rounds of recombination occurred along the selection regime. Therefore, correlated responses in KRHT and CCR in this study are suggested to be the result of either pleiotropy or trait specific, very tightly linked genes, as suggested in QTL studies in another species (Morgan & Mackay, 2006; Norry et al., 2007, 2008). Polymorphic chromosome inversions are not an issue in this case because all of the cytologically detectable inversions are highly polymorphic in the population of Chumbicha (Soto et al., 2010), one of the two populations crossed to set up our base population. This polymorphic population in Chumbicha was selected in previous studies where KRHT and CCR did not changed as a correlated response to selection on each one of these traits (Bertoli et al., 2010; Sambucetti et al., 2010), indicating that both KRHT and CCR are not linked by polymorphic inversions.

Temperature sensitivity in mating physiology can operate via diverse warmth sensors involving multiple systems relevant in different contexts (Miwa et al., 2018; Ni et al., 2013). In *Drosophila*, copulatory success declines with increasing temperature above 26°C even if vigorous courtship attempts take place in males, with no copulation above 36°C (Miwa et al., 2018). Recently, Miwa et al. (2018) found that the artificial activation of warmth‐sensitive neurons (“hot cells”) in some sensorial organs of females precludes copulation even at permissive temperatures below 32°C. In addition, mutational loss of the GR28b.d thermoreceptor protein causes females to copulate even at 36°C (Miwa et al., 2018). Further, multiple molecules can mediate behavioral responses to stress temperatures, facilitating independent tuning of distinct thermosensory responses (Ni et al., 2013), perhaps affecting also KRHT and CCR as correlated responses to thermal sexual selection. Although the *gr28b.d* gene is not included within QTLs for KRHT and CCR in *D. melanogaster* (Norry et al., 2008), complexity from epistatic interactions could not be ruled out.

In correlated selection responses, heat‐hardening treatment after selection had a significant effect only for heat‐selected S lines in this study, increasing KRHT (Figure 3; Table 2), further suggesting that heat sensitivity is implicated in the selection response. Regarding cold‐hardening, the pretreatment improved CCR with similar magnitude in all S and C lines (Figure 4; Table 2), indicating that such a plastic response of cold sensitivity, was largely independent of our selection regimen. These results are consistent with the suggestion that plastic responses are larger for lower than for upper thermal limits (Overgaard, Kristensen, Mitchell, & Hoffmann, 2011; Schou, Mouridsen, Sørensen, & Loeschcke, 2017; Sgrò, Terblanche, & Hoffmann, 2016). The capacity of *Drosophila* to heat harden associates with low rates of heat‐shocked protein synthesis (Johnson et al., 2009), and selection for heat resistance usually reduces Hsp70 expression (reviewed in Sørensen, Kristensen, & Loeschcke, 2003). The lack of a hardening effect on KRHT in C lines could depend on the heat‐hardening time used in this study, but the difference observed between S and C lines was more evident after applying a single heat‐hardening treatment (Figure 3). It is interesting that plasticity in KRHT (i.e., plasticity for upper thermal limits) changed as a correlated response to selection on mating success at high temperature, which is assumed to be a direct target of thermal selection in warm environments. Beneficial effects of heat hardening were also recently found improving short‐term mating success at high temperature in *D. melanogaster* (Stazione et al., 2019). This plastic response is of ecological relevance in small insects living in fluctuating thermal environments, where previous exposures to a mild heat stress could improve mating success at elevated temperature. In addition, previous exposure to heat and cold stress also increases the ability to locate food and breeding resources in the field (Kristensen et al., 2007; Loeschcke & Hoffmann, 2007). As global climatic conditions are changing more rapidly than previously predicted (IPCC, 2018), the persistence of animal populations exposed to climatic stress will often depend on such above‐mentioned responses of phenotypic plasticity in addition to evolutionary responses (Deutsch et al., 2008; Franks & Hoffmann, 2012; Huey et al., 2012; Kingsolver et al., 2011).

Our present results show for the first time a clear‐cut relationship between mating success under heat stress and CCR, with S lines being more cold sensitive than C lines (i.e., a longer CCR time after selecting for mating success at 33°C). In addition, selection on mating success at high temperature revealed an otherwise hidden, indirect *trade‐off* association between KRHT and CCR, which was previously suggested by QTL studies but not visualized across artificial selection experiments on KRHT and CCR in lowland populations. This result could have implications for our understanding of the complexity of genetic correlations between traits of thermal adaptation.

## CONFLICT OF INTEREST

The authors have no conflict of interest to declare.

## AUTHOR CONTRIBUTIONS

P. Sambucetti and F. M. Norry designed the study; L. Stazione and F.H. Gomez carried out the laboratory work; L. Stazione, P. Sambucetti, and F.H. Gomez did the data analyzes; L. Stazione, P. Sambucetti, and F. M. Norry contributed equallly to write the manuscript. All contributing authors have approved this work for publication.

## Data Availability

https://doi.org/10.6084/m9.figshare.11549532.
