# Supplementary figures and images for: Heat knockdown resistance and chill‐coma recovery as correlated responses to selection on mating success at high temperature in Drosophila buzzatii
Source: Ecol Evol. 2020 Feb 6;10(4):1998–2006. doi: 10.1002/ece3.6032 (PMC7042739; doi:10.1002/ece3.6032)

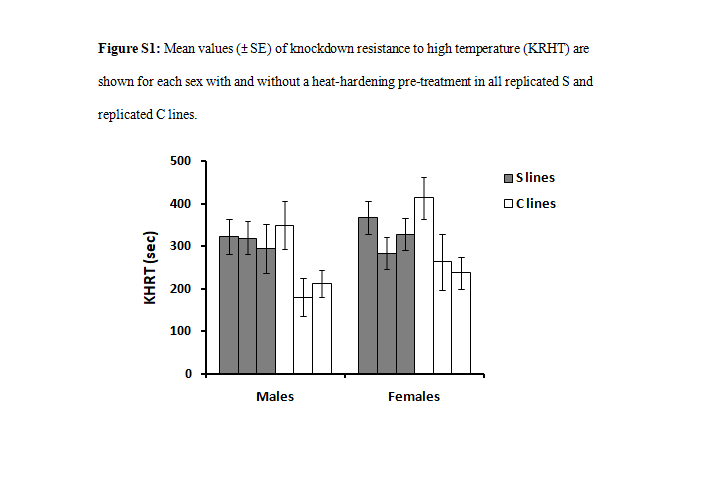

Supplement: Supplementary file 1 [file ECE3-10-1998-s001.tiff]

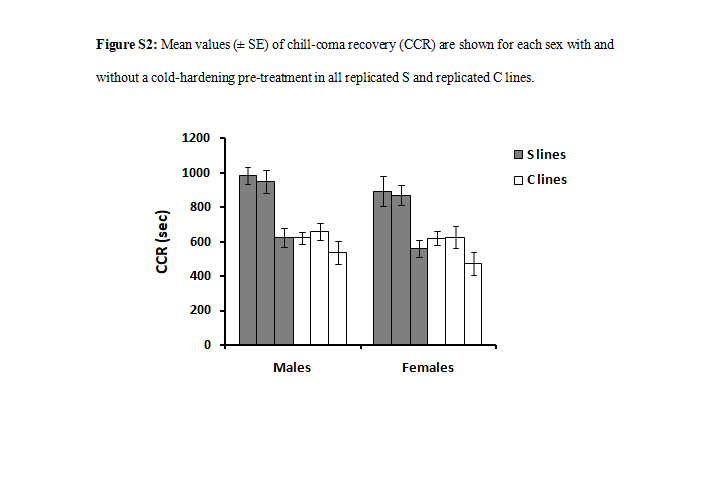

Supplement: Supplementary file 2 [file ECE3-10-1998-s002.tiff]
