# Supplementary material for: Heat knockdown resistance and chill‐coma recovery as correlated responses to selection on mating success at high temperature in Drosophila buzzatii
Source: Ecol Evol. 2020 Feb 6;10(4):1998–2006. doi: 10.1002/ece3.6032 (PMC7042739; doi:10.1002/ece3.6032)
Supplement: Supplementary file 3 [file ECE3-10-1998-s003.docx]

**Table S1:** Mean number of mating flies, standard errors (SE) and number of mating cages (n) at 25°C (A) and 33°C (B) averaged over total mating cages is shown for each sex in each replicates S and C lines.

A

|  |  |  |  | |  |  |  |  |  |
| --- | --- | --- | --- | --- | --- | --- | --- | --- | --- |
| ***Males*** | n | Mean | *S.E* | |  | ***Females*** | n | Mean | *S.E* |
| S1 | 6 | 7.83 | 1.62 | |  | S1 | 6 | 5.50 | 1.65 |
| S2 | 7 | 7.00 | 1.50 | |  | S2 | 7 | 6.14 | 1.14 |
| S3 | 6 | 6.50 | 1.31 | |  | S3 | 6 | 4.50 | 1.34 |
| C1 | 6 | 4.83 | 1.11 | |  | C1 | 6 | 6.67 | 1.56 |
| C2 | 5 | 5.00 | 1.14 | |  | C2 | 5 | 6.40 | 1.25 |
| C3 | 8 | 3.38 | 0.42 | |  | C3 | 8 | 5.63 | 0.68 |
| B |  |  |  |  | |  |  |  |  |
|  |  |  |  | |  |  |  |  |  |
| ***Males*** | n | Mean | *S.E* | |  | ***Females*** | n | Mean | *S.E* |
| S1 | 8 | 8.38 | 1.32 | |  | S1 | 8 | 7.75 | 1.50 |
| S2 | 7 | 7.57 | 1.60 | |  | S2 | 7 | 6.85 | 1.38 |
| S3 | 8 | 7.75 | 1.18 | |  | S3 | 8 | 8.25 | 1.06 |
| C1 | 7 | 3.85 | 0.8 | |  | C1 | 7 | 4.57 | 1.00 |
| C2 | 8 | 3.86 | 0.76 | |  | C2 | 8 | 2.42 | 0.37 |
| C3 | 8 | 3.38 | 0.98 | |  | C3 | 8 | 4.75 | 1.08 |
|  |  |  |  | |  |  |  |  |  |
