# Supplementary material for: Heat knockdown resistance and chill‐coma recovery as correlated responses to selection on mating success at high temperature in Drosophila buzzatii
Source: Ecol Evol. 2020 Feb 6;10(4):1998–2006. doi: 10.1002/ece3.6032 (PMC7042739; doi:10.1002/ece3.6032)
Supplement: Supplementary file 4 [file ECE3-10-1998-s004.docx]

**Table S2:** Mean values, standard error (SE) and sample size (n) of knockdown resistance to high temperature (KRHT) for each sex without (A) and with (B) a heat-hardening pre-treatment in each replicate S and C lines.

A

|  |  |  |  |  |  |  |  |  |
| --- | --- | --- | --- | --- | --- | --- | --- | --- |
| ***Males*** | n | Mean | *S.E* |  | ***Females*** | n | Mean | *S.E* |
| S1 | 50 | 323.4 | 41.00 |  | S1 | 53 | 367.92 | 38.98 |
| S2 | 46 | 319.56 | 38.69 |  | S2 | 52 | 283.27 | 37.43 |
| S3 | 44 | 294.71 | 58.16 |  | S3 | 54 | 328.89 | 38.07 |
| C1 | 49 | 348.73 | 56.37 |  | C1 | 40 | 414.00 | 49.56 |
| C2 | 38 | 18.00 | 45.45 |  | C2 | 37 | 263.33 | 65.72 |
| C3 | 50 | 211.80 | 31.74 |  | C3 | 42 | 237.14 | 38.10 |
| B |  |  |  |  |  |  |  |  |
|  |  |  |  |  |  |  |  |  |
| ***Males*** | n | Mean | *S.E* |  | ***Females*** | n | Mean | *S.E* |
| S1 | 22 | 298.63 | 57.87 |  | S1 | 20 | 373.50 | 70.14 |
| S2 | 23 | 371.74 | 76.02 |  | S2 | 24 | 426.25 | 67.03 |
| S3 | 23 | 465.65 | 85.60 |  | S3 | 26 | 467.31 | 71.50 |
| C1 | 23 | 414.78 | 71.56 |  | C1 | 33 | 402.73 | 61.27 |
| C2 | 24 | 212.50 | 42.58 |  | C2 | 26 | 320.77 | 56.16 |
| C3 | 28 | 157.51 | 48.71 |  | C3 | 29 | 190.34 | 35.59 |
|  |  |  |  |  |  |  |  |  |
