# Supplementary material for: Heat knockdown resistance and chill‐coma recovery as correlated responses to selection on mating success at high temperature in Drosophila buzzatii
Source: Ecol Evol. 2020 Feb 6;10(4):1998–2006. doi: 10.1002/ece3.6032 (PMC7042739; doi:10.1002/ece3.6032)
Supplement: Supplementary file 5 [file ECE3-10-1998-s005.docx]

**Table S3:** Mean values, standard error (SE) and sample size (n) of chill-coma recovery (CCR) for each sex without (A) and with (B) a cold-hardening pre-treatment in each replicate S and C lines.

A

|  |  |  |  |  |  |  |  |  |
| --- | --- | --- | --- | --- | --- | --- | --- | --- |
| ***Males*** | n | Mean | *S.E* |  | ***Females*** | n | Mean | *S.E* |
| S1 | 58 | 983.79 | 51.53 |  | S1 | 49 | 892.41 | 86.56 |
| S2 | 42 | 949.12 | 65.79 |  | S2 | 40 | 869.43 | 59.16 |
| S3 | 22 | 623.58 | 55.28 |  | S3 | 21 | 557.81 | 47.67 |
| C1 | 36 | 621.07 | 36.03 |  | C1 | 27 | 618.50 | 42.17 |
| C2 | 39 | 659.82 | 49.18 |  | C2 | 47 | 625.96 | 64.12 |
| C3 | 26 | 535.55 | 67.42 |  | C3 | 17 | 469.90 | 67.38 |
|  |  |  |  |  |  |  |  |  |
| B |  |  |  |  |  |  |  |  |
| ***Males*** | n | Mean | *S.E* |  | ***Females*** | n | Mean | *S.E* |
| S1 | 50 | 435.34 | 31.47 |  |  | 56 | 333.91 | 21.22 |
| S2 | 44 | 634.36 | 72.45 |  | S2 | 41 | 573.20 | 56.49 |
| S3 | 54 | 508.72 | 49.86 |  | S3 | 58 | 404.33 | 37.66 |
| C1 | 40 | 461.83 | 49.30 |  | C1 | 35 | 268.83 | 34.80 |
| C2 | 46 | 316.37 | 27.07 |  | C2 | 58 | 299.69 | 25.43 |
| C3 | 48 | 322.88 | 32.48 |  | C3 | 48 | 308.17 | 33.74 |
|  |  |  |  |  |  |  |  |  |
